# Higher aortic–brachial pulse wave velocity ratio is associated with large artery atherosclerosis–related ischemic stroke

**DOI:** 10.3389/fneur.2026.1781281

**Published:** 2026-07-15

**Authors:** Kamil Kowalczyk, Mariusz Kwarciany, Krzysztof Narkiewicz, Bartosz Karaszewski, Dariusz Ga̧secki

**Affiliations:** 1 Department of Adult Neurology, Medical University of Gdańsk, Gdańsk, Poland; 2 Department of Hypertension and Diabetology, Medical University of Gdańsk, Gdańsk, Poland

**Keywords:** aortic–brachial pulse wave velocity ratio, arterial stiffness, ischemic stroke, large artery atherosclerosis, pulse wave velocity

## Abstract

**Background:**

Hypertension is one of the main modifiable risk factors for ischemic stroke related to large artery atherosclerosis. Arterial stiffness indices may improve risk stratification beyond blood pressure (BP) measurement alone. Increased pulse wave velocity (PWV) is a risk and prognostic factor for ischemic stroke (IS) and is associated with carotid artery atherosclerosis. We investigated whether the aortic–brachial pulse wave velocity (PWV) ratio differs between stroke subtypes and whether it is independent of mean blood pressure (MBP) in ischemic stroke patients.

**Methods:**

We measured carotid-radial (cr-PWV) and carotid-femoral PWV (cf-PWV) on day 6 of hospitalization in 188 IS subjects. PWV ratio was calculated as cf-PWV/cr-PWV. The etiology of stroke was determined following appropriate diagnostic investigations. Stroke etiology was determined according to TOAST classification. Associations between PWV ratio, stroke subtype, and MBP were analyzed using uni- and multivariate models.

**Results:**

Forty-one patients (21.8%) had stroke related to large artery atherosclerosis (LAA). The PWV ratio was higher in LAA compared with non-LAA stroke [1.26 (1.09–1.58) vs. 1.10 (0.95–1.36), *p* < 0.01], remaining significant after adjustment (OR = 3.40, 95%CI = 1.18–9.79, *p* = 0.02). PWV ratio was not associated with MBP (*p* = 0.79).

**Conclusions:**

PWV ratio is elevated in LAA-related ischemic stroke and was not significantly associated with BP, supporting its potential role as a vascular risk marker.

## 1 Introduction

Hypertension is one of the leading modifiable risk factors for ischemic stroke related to large artery atherosclerosis (LAA) (1). Arterial stiffness indices may improve risk stratification beyond blood pressure (BP) measurement alone. Arterial stiffness is an independent risk factor for cardiovascular and cerebrovascular events, including ischemic stroke (2, 3). It also predicts the outcome of ischemic stroke, with stiffer arteries linked to a poorer prognosis (4–6). Elevated arterial stiffness is a strong indicator of intima-media thickness, which is a marker of atherosclerosis, including carotid arteries (7). Several indices are used to assess local, regional, and systemic arterial stiffness, including but not limited to central pulse pressure, pulse wave velocity, carotid distensibility, or carotid compliance. However, pulse wave velocity (PWV) is widely recognized as the gold standard for assessing arterial stiffness (8).

Only a limited number of studies have investigated the difference in arterial stiffness between the etiological subtypes of ischemic stroke, with the results being inconclusive. One study uncovered that patients with the lacunar subtype of ischemic stroke tended to exhibit higher levels of arterial stiffness in comparison to those with ischemic stroke induced by large-artery atherosclerosis (LAA) or cardioembolism (9). On the other hand, another study found no difference in arterial stiffness between ischemic stroke arising from large-artery atherosclerosis and cardio-embolic infarction (10).

There is a physiological stiffness gradient between the aorta and peripheral muscular arteries due to the aorta's high elasticity. With increasing age, aortic stiffness rises while there is only minimal alteration in peripheral stiffness (11). During the aging process, it is typical for the aorta to become stiffer than the peripheral muscular arteries. This can result in an equalization or even a reversal of the stiffness gradient, referred to as stiffness mismatch (12). Aortic-brachial PWV ratio has been shown to be associated with increased mortality in the dialysis population (12). Additionally, it has been linked to cardiovascular events (13), as well as age and body hydration status in patients with end-stage renal disease (14).

However, the potential usefulness of the aortic-brachial stiffness ratio as a tool to assess arterial stiffness may be limited by its dependence on blood pressure (BP) in some populations (15). Indeed, research has demonstrated that the ratio of aortic-brachial stiffness is contingent on pressure levels in healthy individuals, while it remains uncorrelated with blood pressure in patients with renal dysfunction, hypertension, and type 2 diabetes (14–17). It remains unverified whether aortic-brachial stiffness ratio correlates with blood pressure in people suffering from ischemic stroke. In the analyses, we included mean blood pressure (MBP) as a BP parameter because it is the most important physiological variable influencing arterial stiffness. As MBP increases, the vessels stiffen, but in a non-linear manner. Consequently, the measured stiffness value is dependent on MBP, a factor that must be considered when interpreting the results. Conversely, PP functions as an indirect indicator of large artery stiffness since it is affected by the compliance of the large arteries, in conjunction with the stroke volume and the influence of reflected pressure waves (18).

It was hypothesized that PWV ratio would differ according to the etiology of ischemic stroke. The study aimed to compare PWV ratio among subjects with ischemic stroke, categorized by etiology, and to assess PWV ratio's pressure independence in ischemic stroke patients.

## 2 Materials and methods

The first author had full access to all the data in the study and takes responsibility for its integrity and the data analysis.

### 2.1 Study participants and data collection

Adult patients who suffered from acute ischemic stroke were selected for this study. They were admitted to the Department of Adult Neurology, Stroke Unit in Gdańsk, Poland, within 24 h post the onset of symptoms, during the years 2008–2015. We excluded patients who had a pre-stroke modified Rankin score above 1 and those who had atrial fibrillation during the tonometry session since it is considered a contraindication for applanation tonometry. Prior inclusion in the study, informed written consent was obtained from all patients deemed capable of giving consent. Patients who were unable to give informed consent, for example, due to a severe clinical condition or aphasia, were also included in the study, as, due to the non-invasive nature of the procedures performed, the potential benefits outweighed the possible risks. Clinical data, including medical history and cardiovascular risk factors, were systematically collected upon admission using a standardized protocol. Hypertension, diabetes mellitus, and dyslipidemia were defined based on a documented pre-stroke diagnosis or the use of relevant pharmacological treatment before admission. Information regarding pre-stroke antihypertensive therapy was obtained from a detailed review of outpatient medical records and confirmed through interviews with patients or their caregivers. Combination therapy defined as the use of two or more antihypertensive agents. Patients received suitable medical care, and standard diagnostic procedures were carried out to determine the cause of the ischemic stroke following the Trial of ORG 10172 in Acute Stroke Treatment (TOAST) classification (19). The study was approved by the Bioethics Committee for Scientific Research and performed in compliance with the Helsinki Declaration.

### 2.2 Pulse wave velocity and blood pressure measurements

PWV was measured with applanation tonometry by trained medical professionals (KK, MK, DG) using the SphygmoCor^®^ device (AtCor Medical Pty Ltd., Sydney, Australia) on ischemic stroke patients on day 6 (±2) of hospitalization, only in patients who met the criteria for clinical stability: (1) full consciousness and the ability to maintain a supine position for at least 10 min; (2) hemodynamic stability, defined as the absence of intravenous vasopressors or vasodilators; and (3) no cardiac arrhythmias (e.g., atrial fibrillation) that could interfere with the applanation tonometry signal. Following expert consensus recommendations, all measurements were carried out under standardized conditions (8). In this study, carotid-radial PWV (cr-PWV) and carotid-femoral PWV (cf-PWV) were acquired by sequentially recording pulse on the radial and carotid artery or femoral and carotid artery, respectively. The PWV values were then determined by calculating the transit time between the two arterial sites, measured in relation to the R wave of the electrocardiogram (ECG). The pulse transit time was obtained by subtracting the time between the proximal pulse and the ECG from the time between the distal pulse and the ECG. The first segment of the pressure waveform served as a reference point (8). The highest quality measurement was chosen after reviewing the waveforms and assessing the embedded quality indices. The calculation of PWV ratio followed as cf-PWV/cr-PWV. Blood pressure and heart rate (HR) were measured with the Omron HEM-705C (Omron Healthcare Co., Ltd., Kyoto, Japan) oscillometric device after at least a 10-min rest, with the patient in the supine position, immediately before performing applanation tonometry. The last two measurements were averaged and used in further analyses.

### 2.3 Statistical analysis

Baseline data were expressed as the median (interquartile range) or number (percentage). Median (interquartile range) was used to express PWV values. The comparison of PWV values by stroke etiology was calculated through the Kruskal–Wallis *H* test with Dunn's *post hoc* test for multiple comparisons. The etiological group with the highest median PWV ratio value was selected, and the remaining etiological groups were combined and compared to the first group. Univariate comparisons between the two cohorts were conducted using the Mann–Whitney *U* test for quantitative variables or chi-squared test for categorical variables. Multivariable comparisons were performed utilizing logistic regression analysis, which integrated variables with a *p*-value ≤ 0.1 in the univariable analysis. To investigate the relationship between MBP and cr-PWV, cf-PWV or PWV ratio, a linear regression analysis was carried out. Each model underwent assessment for assumptions of linearity, normality of residuals, collinearity, homoscedasticity, and outliers. Since the residuals were non-normally distributed, the dependent variable was subjected to logarithmic transformation. To control for known or potential confounders (age, sex, cardiovascular disease, body mass index, fasting glucose, and heart rate), multiple linear regression was carried out. A two-tailed *p*-value < 0.05 was deemed statistically significant. The statistical analysis was conducted by KK through STATISTICA version 13 (TIBCO, Palo Alto, California, United States).

## 3 Results

### 3.1 Study cohort

Among the 188 patients recruited, the mean age was 62.4 ± 12.0 years with 66% being male. The National Institutes of Health Stroke Scale (NIHSS) score was 5 (3–8). Of the total, 41 (21.8%) suffered from stroke due to LAA, 44 (23.4%) due to small-vessel disease (SVD), 31 (16.5%) suffered from cardioembolism, 61 (32.4%) stroke of undetermined cause and 11 (5.9%) experienced stroke of other determined cause.

### 3.2 Pulse wave velocity by stroke etiology

In the patient cohort, there were differences in the cf-PWV, cr-PWV, and PWV ratio among ischemic stroke patients based on etiology [H (4, *N* = 188) = 11.6, *p* = 0.02; H (4, *N* = 188) = 12.3, *p* = 0.02; H (4, *N* = 188) = 13.0, *p* = 0.01; respectively]. Patients with stroke due to small-vessel disease had the highest median cf-PWV, patients with stroke of other determined causes had the highest median cr-PWV, and patients with stroke due to LAA had the highest PWV ratio values (Table 1). After the *post-hoc* analysis, it was discovered that patients with LAA-stroke had higher cf-PWV levels than those with stroke of unknown cause (*z* = 3.18, *p* = 0.01). Furthermore, cr-PWV levels were higher in patients with SVD-stroke compared to those with cardioembolic stroke (*z* = 3.36, *p* = 0.01). Additionally, PWV ratio was observed to be higher in patients with LAA-stroke as compared to those with stroke of unknown cause (*z* = 3.52, *p* < 0.01). Patients who experienced a stroke due to a cause other than LAA were pooled for further analysis.

**Table 1:** Comparison of cf-PWV, cr-PWV, and PWV ratio by stroke etiology.

Parameters	LAA (*n* = 41)	SVD (*n* = 44)	CE (*n* = 31)	SOC (*n* = 11)	SUC (*n* = 61)	*p*-Value
cf-PWV, m/s	10.5 (8.8–13.0)	10.9 (8.2–12.2)	9.3 (7.8–12.3)	10.0 (8.2–12.4)	8.9 (8.0–10.9)	0.02
cr-PWV, m/s	8.7 (7.6–9.4)	9.0 (8.5–9.9)	7.9 (6.9–9.3)	9.1 (7.8–10.0)	8.4 (7.9–9.5)	0.02
PWV ratio	1.26 (1.09–1.58)	1.11 (0.96–1.39)	1.14 (1.00–1.48)	1.10 (0.92–1.30)	1.08 (0.93–1.30)	0.01

CE, cardioembolism; cf-PWV, carotid-femoral pulse wave velocity; cr-PWV, carotid-radial pulse wave velocity; LAA, large artery atherosclerosis; PWV, pulse wave velocity; SOC, stroke of other determined cause; SUC, stroke of undetermined cause; SVD, small-vessel disease.

### 3.3 Baseline characteristics

It was observed that the male gender was more predominant in the LAA group as compared to the non-LAA group. The LAA group also had higher levels of C-reactive protein (CRP) and systolic blood pressure (SBP) and were more likely to have partial anterior circulation syndrome and less likely to have lacunar circulation syndrome. Additionally, the LAA group tended to smoke more often and have a higher initial NIHSS score. There were no significant differences between the LAA and non-LAA groups regarding age, hypertension, diabetes, dyslipidemia, and coronary heart disease. Similarly, body mass index, fasting glucose levels, and the frequency of intravenous thrombolysis were comparable. Most importantly, the groups did not differ in the number of antihypertensives taken before or during hospitalization. Furthermore, no disparities were found in the prevalence of pre-stroke combination therapy (Table 2).

**Table 2:** Baseline characteristics of participants according to stroke cause (LAA vs. non-LAA).

Parameters	Overall	LAA (*n* = 41)	Non-LAA (*n* = 147)	*p*-Value
Sex				
Male	125 (66.5)	33 (80.5)	92 (62.6)	0.03
Female	63 (33.5)	8 (19.5)	55 (37.4)	
Age, years	63.0 (56.0–70.0)	64.0 (57.0–73.0)	62.0 (55.0–69.0)	0.13
Medical history			
Hypertension	133 (70.7)	30 (73.2)	103 (70.1)	0.70
Diabetes	96 (51.1)	24 (58.5)	72 (49.0)	0.28
Dyslipidaemia	165 (87.8)	39 (95.1)	126 (85.7)	0.18
Smoking (past or current)	98 (52.1)	26 (63.4)	72 (49.0)	0.10
Coronary heart disease	48 (25.5)	13 (31.7)	35 (23.8)	0.31
Body mass index, kg/m^2^	28.2 (25.6–31.1)	27.3 (24.3–31.2)	28.3 (25.6–31.1)	0.48
Number of antihypertensives before hospitalization	1.0 (0.0–2.0)	0.5 (1.0–2.0)	1.0 (0.0–2.0)	0.95
Combination antihypertensive therapy before hospitalization	48 (25.5)	10 (24.4)	38 (25.9)	0.87
Stroke characteristics			
Initial NIHSS score	5 (3–8)	6 (4–12)	5 (3–8)	0.05
Initial glucose level, mg/dL	116 (103.0–152.0)	112.0 (100.0–134.0)	117 (105.0–158.0)	0.11
Fasting glucose level, mg/dL	100.0 (92.0–117.0)	102.0 (91.0–120.0)	99.0 (92.0–117.0)	0.63
Initial CRP level, mg/L	2.82 (1.38–6.40)	3.70 (1.90–9.60)	2.59 (1.27–4.80)	0.02
Intravenous thrombolysis	56 (29.8)	11 (26.8)	45 (30.6)	0.64
Number of antihypertensives during hospitalization	1.0 (1.0–2.0)	1.5 (1.0–2.0)	1.0 (1.0–2.0)	0.82
Haemodynamic parameters			
Systolic blood pressure, mm Hg	137.0 (124.0–155.0)	148.0 (133.0–168.0)	134.0 (123.0–151.0)	0.01
Diastolic blood pressure, mm Hg	80.0 (72.0–89.0)	81 (76–89)	79.5 (72.0–89.0)	0.56
Heart rate, beats per minute	65.0 (59.0–71.0)	65.0 (59.0–70.0)	65.0 (59.0–71.0)	0.89
cf-PWV, m/s	9.8 (8.2–12.2)	10.5 (8.8–13.0)	9.5 (8.0–11.9)	0.01
cr-PWV, m/s	8.7 (7.8–9.6)	8.7 (7.6–9.4)	8.7 (7.9–9.7)	0.54
PWV ratio	1.14 (0.97–1.40)	1.26 (1.09–1.58)	1.10 (0.95–1.36)	< 0.01
Stroke OCSP classification				0.05
TACS	17 (9.0)	4 (9.8)	13 (8.8)	0.90
PACS	77 (41.0)	23 (56.1)	54 (36.7)	0.03
LACS	51 (27.1)	5 (12.2)	46 (31.3)	0.01
POCS	43 (22.9)	9 (22.0)	34 (23.1)	0.87

Results are given as n (%) or median (interquartile range).

cf-PWV, carotid-femoral pulse wave velocity; cr-PWV, carotid-radial pulse wave velocity; CRP, C-reactive protein; LAA, large artery atherosclerosis; LACS, lacunar syndrome; NIHSS, National Institutes of Health Stroke Scale; OCSP, the Oxfordshire Community Stroke Project; PACS, partial anterior circulation syndrome; POCS, posterior circulation syndrome PWV, pulse wave velocity; TACS, total anterior circulation syndrome.

### 3.4 Pulse wave velocity in LAA vs. non-LAA stroke patients

In the univariate analysis, patients with LAA showed elevated levels of cf-PWV [10.5 (8.8–13.0) m/s vs. 9.5 (8.0–11.9) m/s, *p* = 0.01] and PWV ratio [1.26 (1.09–1.58) vs. 1.10 (0.95–1.36), *p* < 0.01] when compared to non-LAA patients. However, there were no significant differences in cr-PWV between the groups [8.7 (7.6–9.4) m/s vs. 8.7 (7.9–9.7) m/s, *p* = 0.54]. In the multivariable analysis,incorporating confounders such as sex, smoking status, baseline NIHSS score, and systolic blood pressure, cf-PWV exhibited no significant variation amid the subgroups [adjusted OR (95% CI), 1.15 (0.98–1.36), *p* = 0.10; Table 3, Model 1]. Conversely, PWV ratio retained a significant association with stroke due to LAA [adjusted OR (95% CI), 3.40 (1.18–9.79), *p* = 0.02; Table 3, Model 2].

**Table 3:** Multivariate logistic regression analysis models for comparisons between LAA and non-LAA stroke.

Models	Parameters	OR	95% CI	*p*- Value
Model 1	Sex	2.76	0.99–7.73	0.05
	Smoking	2.43	0.98–6.02	0.05
	Initial NIHSS score	1.08	1.00–1.17	0.04
	Initial CRP	1.02	0.99–1.06	0.21
	SBP	1.01	0.99–1.03	0.18
	cf-PWV	1.15	0.98–1.36	0.10
Model 2	Sex	3.19	1.12–8.99	0.03
	Smoking	2.35	0.97–5.73	0.06
	Initial NIHSS score	1.08	1.00–1.17	0.06
	Initial CRP	1.02	0.99–1.06	0.18
	SBP	1.02	1.00–1.04	0.04
	PWV ratio	3.40	1.18–9.79	0.02

95% CI, 95% confidence interval; cf-PWV, carotid-femoral pulse wave velocity; CRP, C-reactive protein; LAA, large artery atherosclerosis; NIHSS, National Institutes of Health Stroke Scale; OR, odds ratio; SBP, systolic blood pressure.

To further investigate the impact of age, we performed an age-stratified analysis based on the median age of 63 years. In the younger subgroup (< 63 years, *n* = 92), patients with LAA stroke (*n* = 16) presented with a significantly higher PWV ratio compared to those with other stroke etiologies [median (IQR): 1.22 (1.15–1.48) vs. 1.01 (0.84–1.16); *p* = < 0.001]. In contrast, in the older subgroup (≥63 years, *n* = 96), the difference in PWV ratio between LAA and non-LAA patients did not reach statistical significance [1.31 (1.06–1.64) vs. 1.24 (1.08–1.55); *p* = 0.445].

### 3.5 Dependence of PWV ratio on blood pressure

**Figure 1:**
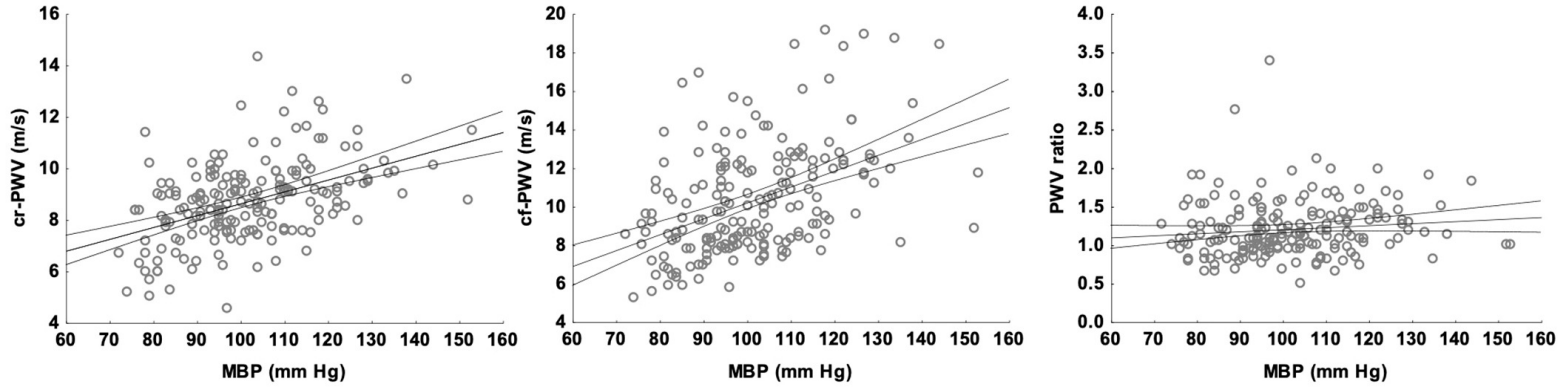
Relationship between mean blood pressure (MBP) and carotid-femoral pulse wave velocity (cf-PWV), carotid-radial pulse wave velocity (cr-PWV), and aortic-brachial pulse wave velocity ratio (PWV ratio). Dashed lines indicate 95% confidence interval.

In the univariate linear regression analysis, cr-PWV and cf-PWV were significantly associated with MBP (standardized β = 0.4660, *p* < 0.01 and standardized β = 0.4650, *p* < 0.01; respectively); however, there was not such a relationship between PWV ratio and MBP (standardized β = 0.1376, *p* = 0.06). Figure 1 shows relationship between arterial stiffness and MBP, and Figure S1 shows relationship between arterial stiffness and MBP with regression equations and *R*^2^ values after logarithmic transformation. After adjustment for possible confounders, including age, sex, cardiovascular disease, body mass index, fasting glucose, and HR, PWV ratio was not significantly associated with MBP (standardized β = −0.0204, *p* = 0.79; Table 4).

**Table 4:** Multivariate linear regression analysis of the relationship between PWV ratio and MBP considering confounders.

Parameters	Standardized β	Partial *R*^2^	*p*- Value
MBP	−0.0204	0.113	0.79
Age	0.3876	0.095	< 0.01
Sex	−0.1028	0.084	0.17
Coronary heart disease	0.1337	0.118	0.08
BMI	0.0259	0.027	0.72
Fasting glucose	0.3433	0.076	< 0.01
Heart rate	0.0440	0.104	0.56

Model *R*^2^ = 0.377.

BMI, body mass index; MBP, mean blood pressure.

## 4 Discussion

This study is the first to analyze PWV ratio in patients with ischemic stroke. Our research indicates that: (1) PWV ratio is greater in patients with LAA-induced stroke compared to those with non-LAA stroke and (2) in sufferers of ischemic stroke, PWV ratio was not associated with blood pressure.

In our research, we compared PWV ratio values among different etiologies of ischemic stroke. The results indicated that strokes associated with large artery atherosclerosis had the highest ratios. In addition, a *post-hoc* analysis revealed that LAA-stroke patients had a higher PWV ratio compared to those with stroke of other determined cause. Furthermore, when comparing PWV ratios in patients with LAA stroke to those with stroke of other or unknown causes (combined), patients with LAA stroke demonstrated a higher PWV ratio, even after controlling for various confounding factors, including sex, smoking, initial NIHSS score, initial CRP level, and SBP.

While no studies have assessed PWV ratio in ischemic stroke patients, recent reports have shown an association between cf-PWV and carotid artery atherosclerosis, as measured by carotid intima-media thickness, in the general population (20), elders (21, 22), hypertensive (23, 24), and diabetic patients (25). Based on this information, it appears logical that a higher PWV ratio is present in patients with ischemic strokes caused by LAA when compared to those with non-LAA stroke. This may be especially true as cr-PWV typically declines after the age of 50 (26). A lower cr-PWV yields a higher PWV ratio because cr-PWV is in the denominator of the formula calculating PWV ratio. It has been suggested that reduced stiffness of the brachial arteries may be an adaptive change to increased central aortic stiffness (12). However, our findings partially diverge from a separate study which found a greater cf-PWV in lacunar ischemic stroke patients compared to those with stroke caused by LAA, cardioembolism (CE), and stroke of other determined cause (SOC) (9). Our results showed a higher cf-PWV in LAA stroke patients as opposed to those with strokes of undetermined cause, but no significant cf-PWV differences were found between other subgroups of stroke in a *post-hoc* analysis. There may be several reasons for this inconsistency. Firstly, subjects with stroke caused by LAA in our study, as well as lacunar subgroup in the other study displayed higher blood pressure values, which is a known major determinant of PWV (27). Secondly, according to the study by Tuttolomondo et al., patients with lacunar infarct were the oldest, and age is another major determinant of PWV (27). Bearing these factors in mind, it is possible that high cf-PWV may partially relate to factors other than aetiologic subtype, such as age and BP.

Our study reveals that the association between the PWV ratio and LAA stroke etiology is age-dependent. In the age-stratified analysis, the PWV ratio remained a highly significant marker in the younger subgroup (< 63 years), whereas it did not reach statistical significance in older patients. This finding is consistent with the phenomenon of stiffness gradient reversal observed with advancing age. In younger individuals, the peripheral arteries (reflected by cr-PWV) are typically elastic, and an increase in central stiffness (cf-PWV) creates a clear, detectable gradient (high PWV ratio) that points toward LAA pathology. However, as the entire arterial tree stiffens physiologically with age, the ratio between central and peripheral stiffness tends to equalize, which may mask the specific contribution of large artery atherosclerosis to the overall stiffness profile in the elderly (11).

Importantly, our analysis showed that the observed differences in the PWV ratio were not confounded by pharmacological treatment. The number of antihypertensive drugs—both prior to stroke onset and during the subacute phase—was similar across all stroke subtypes. This suggests that the increased arterial stiffness in LAA patients reflects intrinsic structural remodeling of the arterial wall rather than transient effects of medication or acute blood pressure management.

In the present study, the PWV ratio was calculated from measurements obtained on day 6 post-stroke. This time point was chosen to represent the early subacute phase, once clinical and hemodynamic stability had been achieved (i.e., stable blood pressure and no requirement for intravenous therapy). While some studies have observed that PWV values measured more than 3 months after a stroke may be lower than those recorded on day 6, reflecting the resolution of the acute stress response (28), assessment during the early phase remains highly clinically relevant. Indeed, PWV measured in the acute and subacute phases has been shown to be a strong predictor of both early and late prognosis following ischemic stroke (5, 6). Therefore, our findings highlight the diagnostic utility of the PWV ratio during this critical clinical window. Nevertheless, further studies are needed to determine the long-term evolution of the PWV ratio and its predictive value at later stages, such as 3 months post-stroke.

Another important observation from this study is that we did not detect a significant association between PWV ratio and MBP in either univariate or multivariable analyses. Although this finding suggests that PWV ratio may be less dependent on blood pressure than cf-PWV or cr-PWV in patients with ischemic stroke, it should be interpreted cautiously given the modest sample size and the possibility of more complex, non-linear relationships between arterial stiffness and blood pressure. To our knowledge, this is the first study to report this observation in patients with cerebral infarction, although confirmation in larger cohorts is needed. Prior to this study, numerous research reports assessed the BP-independence of PWV ratio. This parameter has been proven to be independent of blood pressure in dialysis patients, as well as patients with an estimated glomerular filtration rate greater than 45 mL/min/1.73 m^2^ who are either hypertensive or have undergone a kidney transplant (as indicated by univariate and multivariate analyses) (16), in patients with renal dysfunction, hypertension, and diabetes (univariate and multivariate analyses) (15, 17), and in end-stage renal disease patients (14). On the other hand, PWV ratio has been shown to be significantly and independently associated with mean arterial pressure in healthy subjects (15). Our finding, in the context of the above results, may have clinical significance, as PWV ratio may be used in high-risk populations to assess vascular status. The difference in the BP dependence of the index between healthy individuals and patients is probably related to vascular changes associated with age and disease processes affecting the large artery walls (15). As people age and develop diseases, the aorta tends to stiffen (29). Several factors contribute to this phenomenon, including changes in the structure of the arterial wall (collagen deposition, elastin loss, medial calcification, non-enzymatic cross-linking between microfibrils) (29).

Importantly, the present study should be considered exploratory and hypothesis-generating due to several potential limitations. First, the study was single-center and retrospective in nature. Consequently, it is susceptible to selection bias, and it becomes impossible to establish a causality. Second, we acknowledge that the relatively small sample size, particularly in the LAA subgroup (*n* = 41), may affect the generalizability of our findings and warrants cautious interpretation. Third, we recognize that combining non-LAA stroke subtypes with distinct pathophysiological mechanisms (cardioembolic, small vessel disease, undetermined, and other etiologies) into a single comparator group introduces substantial clinical and pathophysiological heterogeneity. While this approach reflects real-world clinical practice, it may mask subtle differences in arterial stiffness profiles across specific subtypes, such as cardioembolic or small-vessel disease. This heterogeneity likely reduced the specificity of the observed associations and limits the direct clinical applicability of our findings, making the observed association between PWV ratio and LAA preliminary in nature. Future studies with larger and more balanced cohorts will be essential to evaluate its discriminatory value across individual stroke subtypes and to better define its specificity and potential role in clinical practice.

While our findings suggest that the PWV ratio is a promising marker for LAA etiology, confirmation in larger, more homogeneous cohorts is required to validate its discriminatory power across specific non-LAA subtypes. Fourth, the LAA and non-LAA stroke groups are heterogenous, potentially impacting the interpretation of PWV differences. The LAA group, as expected, had a higher proportion of TACS/PACS syndromes, but also included 12% with LACS, typically linked to small vessel disease. Such overlap is not uncommon in clinical practice due to mixed vascular pathology and limitations in classification systems like TOAST and OCSP, especially when advanced imaging (e.g., computed tomography angiography or magnetic resonance angiography) is not performed in all cases. Moreover, TOAST classification has several limitations, including the fact that the site of the brain infarction is not sufficiently specific to clearly identify the cause of the stroke. Lacunar syndrome may be caused not only by small vessel disease but also by embolism, genetic conditions like CADASIL, or large artery stenosis (30, 31). Prior studies suggest that 8%−20% of lacunar infarcts may be attributable to large artery disease (32, 33), consistent with our findings (12% patients with lacunar syndrome in our LAA group).The proportion of undetermined strokes in our non-LAA group (41%) is relatively high. However, as previous works have shown, the proportion of undetermined strokes remains substantial even in well-characterized cohorts, which is one of the limitations of TOAST classification. The percentage of patients with ischemic stroke of unknown cause may be up to 40% (34), which even exceeds the percentage of such patients found in our study group (32% in the whole group). In another study, the percentage of patients with undetermined cause of stroke was 39.4% in the whole group and 43% in the non-LAA group (35). The relatively high proportion of patients with undetermined etiology in our group may be also because it was selected and did not include patients with persistent atrial fibrillation as this condition is a contraindication to applanation tonometry. Despite the heterogeneity of the groups and the fact that the distributions of the percentage of TACS/PACS stroke patients in the non-LAA group with no known cardiogenic cause and those with stroke of undetermined etiology are not significantly different from those reported in the literature, they significantly limit the strength of any inference regarding pathogenesis and its relation to arterial stiffness. Given the complexities involved and the limitations of the current study, our findings suggest that the PWV ratio is a promising marker for LAA etiology; however, further research in larger, more homogeneous cohorts is necessary to confirm these observations and validate its discriminatory power across specific non-LAA subtypes.

The lack of statistical significance in the older subgroup (≥63 years) does not necessarily imply a lack of clinical relevance but rather underscores the confounding effect of generalized age-related vascular remodeling. In older subjects, the baseline aortic stiffness is already high across all stroke subtypes, which reduces the discriminatory power of the PWV ratio compared to younger cohorts.

From a clinical perspective, the utility of the PWV ratio remains to be established. Our findings do not support its use as a standalone diagnostic tool for LAA-related stroke; however, the PWV ratio may serve as a useful adjunctive marker, particularly in younger and middle-aged patients in whom identification of large artery atherosclerosis has important implications for secondary prevention. In this population, an elevated PWV ratio may reflect disproportionately increased central arterial stiffness relative to age and raise suspicion of underlying atherosclerotic disease, especially when conventional risk factors or standard diagnostic findings are inconclusive. As such, the PWV ratio may help refine clinical suspicion of LAA, guide the need for more detailed vascular imaging, and support etiological classification in patients with competing stroke mechanisms. In this cohort, PWV ratio was not significantly associated with mean blood pressure, which may enhance its value as a complementary measure of vascular status. Future studies should determine whether PWV ratio also provides prognostic information beyond established clinical and imaging markers.

In conclusion, our findings demonstrate an independent association between stroke due to LAA and higher PWV ratio, whereas no such association was observed for cf-PWV. In addition, the lack of a significant association between PWV ratio and mean blood pressure supports its potential utility as an adjunctive marker of arterial stiffness in this cohort.

## Data Availability

The raw data supporting the conclusions of this article will be made available by the authors, without undue reservation.

## Supplementary material

Figure S1 Relationship between mean blood pressure (MBP) and carotid-femoral pulse wave velocity (cf-PWV), carotid-radial pulse wave velocity (cr-PWV), and aortic-brachial pulse wave velocity ratio (PWV ratio) after logarithmic transformation of the dependent variable. Dashed lines indicate 95% confidence interval.
