# Supplementary figures and images for: Higher aortic–brachial pulse wave velocity ratio is associated with large artery atherosclerosis–related ischemic stroke
Source: Front Neurol. 2026 Jul 15;17:1781281. doi: 10.3389/fneur.2026.1781281 (PMC13416833; doi:10.3389/fneur.2026.1781281)

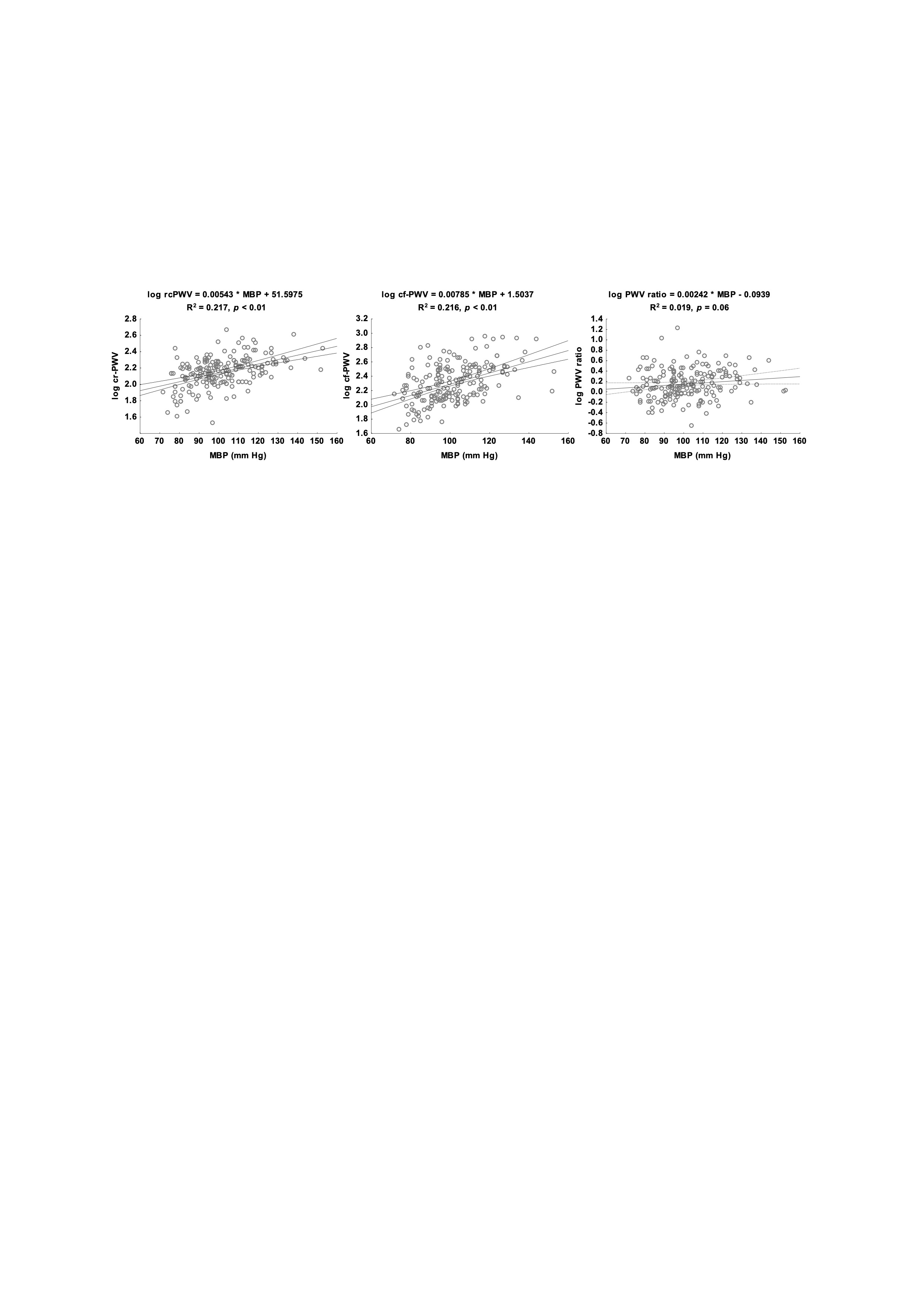

Supplement: Figure S1 — Relationship between mean blood pressure (MBP) and carotid-femoral pulse wave velocity (cf-PWV), carotid-radial pulse wave velocity (cr-PWV), and aortic-brachial pulse wave velocity ratio (PWV ratio) after logarithmic transformation of the dependent variable. Dashed lines indicate 95% confidence interval. [file Image_1.jpeg]
